# Illusory size determines the perception of ambiguous apparent motion

**DOI:** 10.3758/s13423-020-01786-9

**Published:** 2020-08-10

**Authors:** Madeleine Y. Stepper, Cathleen M. Moore, Bettina Rolke, Elisabeth Hein

**Affiliations:** 1 Evolutionary Cognition - Cognitive Science, Department of Psychology, University of Tübingen, Schleichstrasse 4, D - 72076 Tübingen, Germany; 2 University of Iowa, Iowa City, IA USA

**Keywords:** Visual perception, Apparent motion, Object correspondence, Ponzo illusion

## Abstract

The visual system constructs perceptions based on ambiguous information. For motion perception, the correspondence problem arises, i.e., the question of which object went where. We asked at which level of processing correspondence is solved – lower levels based on information that is directly available in the retinal input or higher levels based on information that has been abstracted beyond the input directly available at the retina? We used a Ponzo-like illusion to manipulate the perceived size and separations of elements in an ambiguous apparent motion display. Specifically, we presented Ternus displays – for which the type of motion that is perceived depends on how correspondence is resolved – at apparently different distances from the viewer using pictorial depth cues. We found that the perception of motion depended on the apparent depth of the displays, indicating that correspondence processes utilize information that is produced at higher-level processes.

**Electronic supplementary material:**

The online version of this article (10.3758/s13423-020-01786-9) contains supplementary material, which is available to authorized users.

## Introduction

**Fig. 1:**
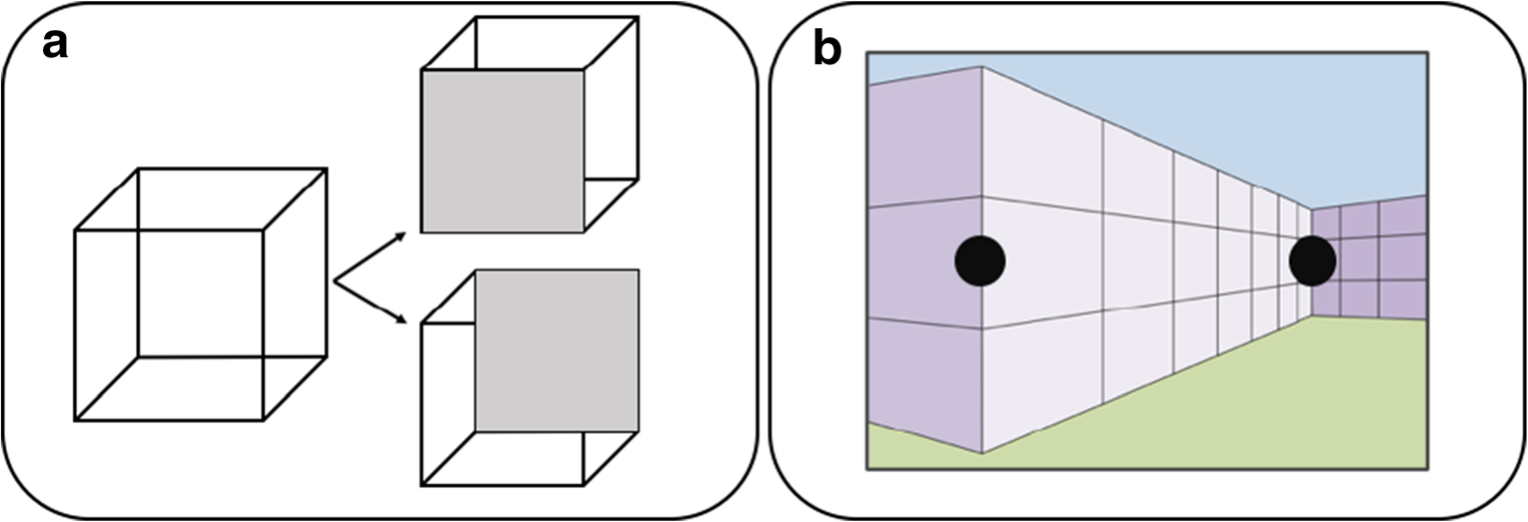
(**a**) Necker cube with its bi-stable percepts regarding the orientation on the left, disambiguated on the right side. (**b**) Ponzo-like size illusion. The element on the right side is perceived as farther away and bigger in size compared to the element on the left side, although both are physically the same size

**“**It’s an illusion” is how we could describe our perception of the three-dimensional world around us. This is because our perception is a constructed representation that is created by our visual system on the basis of ambiguous input information. The information that our visual system receives at the retina is a two-dimensional projection of the three-dimensional environment, which means that it is underdetermined and ambiguous. That our visual system actively resolves this ambiguity is evident in bi-stable perceptions such as our experience of the Necker cube (Necker, 1832; Fig. 1a), which can be perceived as oriented in two different ways, despite no change in retinal input, and in illusions like the Ponzo-like size illusion (sometimes also known as the corridor illusion, Fig. 1b), in which identically sized stimuli are perceived as different sized objects because they are perceived as being at different distances from the viewer. Phenomena like these demonstrate that the interpretation of image-level information, which is what is directly available at the retina, depends on top-down processes that themselves utilize higher-level information (e.g., Kornmeier & Bach, 2012).

**Fig. 2:**
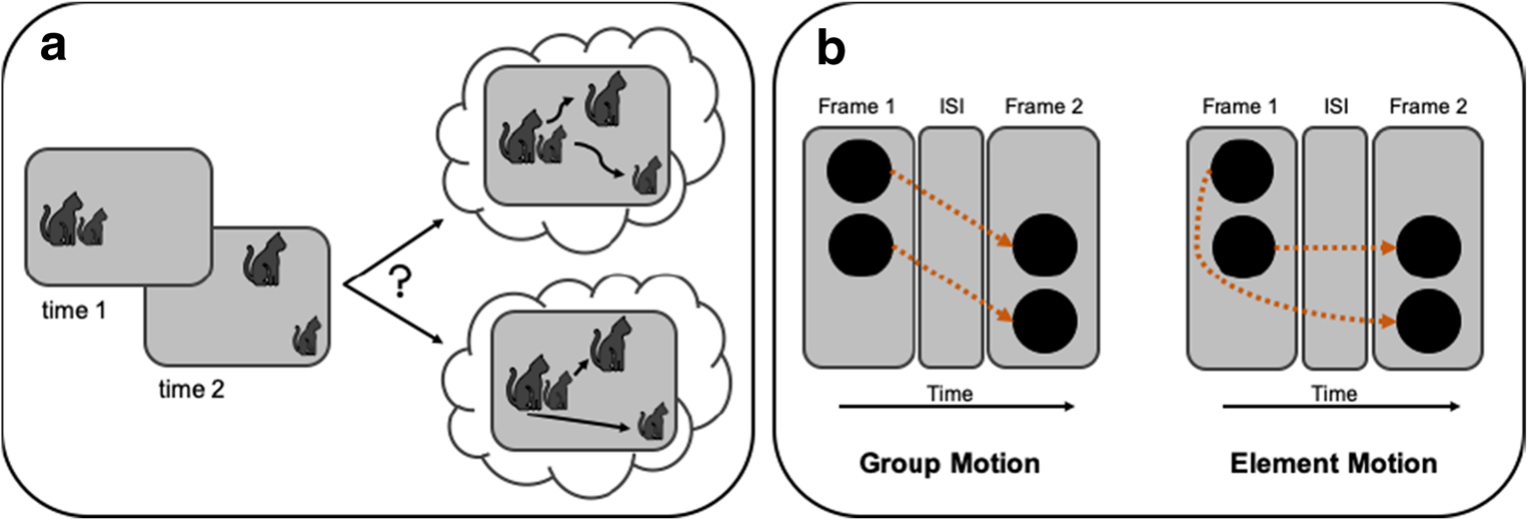
(**a**) Illustration of the correspondence problem with the question of which object went where. Are the cats connected and motion perceived based on their retinal size (upper solution) or based on their distance (lower solution)? (**b**) Vertical version of the Ternus display and the two alternative motion percepts. The two successively presented Ternus frames are separated by a variable interstimulus interval (ISI). They can be perceived as either moving together (group motion) or as one element jumping across the other (element motion)

The challenge of ambiguous input arises not only with static images like those shown in Fig. 1, but also with dynamic input. The identity of objects must be maintained across space and time, even as they become invisible because they are occluded by other objects due to their own or the viewer’s motion. As with the static examples, it is clear that our perception of objects over time depends on active top-down interpretation of ambiguous information. In the case of basic apparent motion, for example, successively presented static stimuli at different locations are perceived as a single object moving from one location to another if – and only if – the time and separation between them is consistent with how objects move in the world (Kolers, 1972; Korte, 1915; Wertheimer, 1912). The perception of objects over time becomes even more complex when, as is typical in natural environments, multiple stimuli are present in given static images – Fig. 2a illustrates the problem. Will motion be perceived based on spatial separation, retinal size, or neither? More generally, the question is how and on the basis of what information does our visual system determine which object went where? This problem, known as the *correspondence problem* (Ullmann, 1979), is a computational challenge because the image-based input is ambiguous (e.g., Dawson, 1991).

To address the question on what information correspondence is based, researchers have used ambiguous apparent motion displays, analogous to the example illustrated in Fig. 2a, in which depending on how correspondence is established, alternative and mutually exclusive motion percepts are experienced. An example of such a display is the Ternus display (Pikler, 1917; Ternus, 1926), which usually consists of two elements horizontally aligned next to each other, shifted by one element position from one frame to the next. For our purpose we created a vertical version of this display, in which two elements were vertically aligned, one above the other (Fig. 2b). Depending on the perceived correspondence between elements across frames, two alternative motion percepts are experienced. In one case, both elements appear to shift together (*group motion*), whereas in the other case, one element appears to “jump” across the other (*element motion*). Which type of motion is perceived – element or group – is therefore a way of assessing how correspondence was resolved. This is why the Ternus display is well suited for investigating the factors that determine correspondence (for an overview, see Hein, 2017; Petersik & Rice, 2006). Studies using Ternus displays have shown that image-level information plays a role in determining correspondence, including the time between frames – the *interstimulus interval* (ISI) – (Navon, 1976; Petersik & Pantle, 1979) and the spatial separation of the elements (Casco, 1990; Navon, 1976; Petersik & Grassmuck, 1981). In particular, the longer the ISI and the smaller the separation of the elements, the more group motion is perceived. In addition, studies have shown that feature information of the elements, such as luminance contrast, color, texture pattern (Hein & Moore, 2012; Petersik & Rice, 2008), and size (Breitmeyer & Ritter, 1986a, 1986b; Casco, 1990; Petersik & Grassmuck, 1981) influence correspondence. Breitmeyer and Ritter (1986a), for example, manipulated the size of the elements and found that larger Ternus elements lead to more group motion percepts compared to smaller ones. Thus, both spatio-temporal information and feature information determines the identity of the elements and how correspondence is resolved to give rise to one motion percept or the other.

While it is clear that feature information plays a role in determining correspondence, it is not clear whether it is feature information at the level of the retinal image or feature information at the level of the perceived object. Because correspondence reflects object identity – i.e., which object went where – it seems likely on functional grounds that it is the perceived feature information of the object that is critical, rather than the image feature. Some evidence consistent with this intuition comes from studies that have shown that the perceptual completion of objects that appear to extend behind occluding surfaces (*amodal completion*) is established before the motion percept is determined (He & Nakayama, 1994; He & Ooi, 1999; Yantis, 1995, Hein & Moore, 2014). He and Nakayama (1994), for example, showed that correspondence can be established on the basis of matching the perceived shapes of perceptually completed surfaces that were occluded by other surfaces, instead of the shapes of the physically visible parts of them. The fact that such information is used to determine correspondence suggests that it takes place at or after a level of visual processing at which amodal completion has taken place. This implies that correspondence can be determined by perceived object identity and does not necessarily have to rely only on image-level features. As this would require a significant rethinking of our understanding of the function of the correspondence process, the goal of the current study was to further investigate the influence of perceived object identity beyond the level of amodal completion processes.

**Fig. 3:**
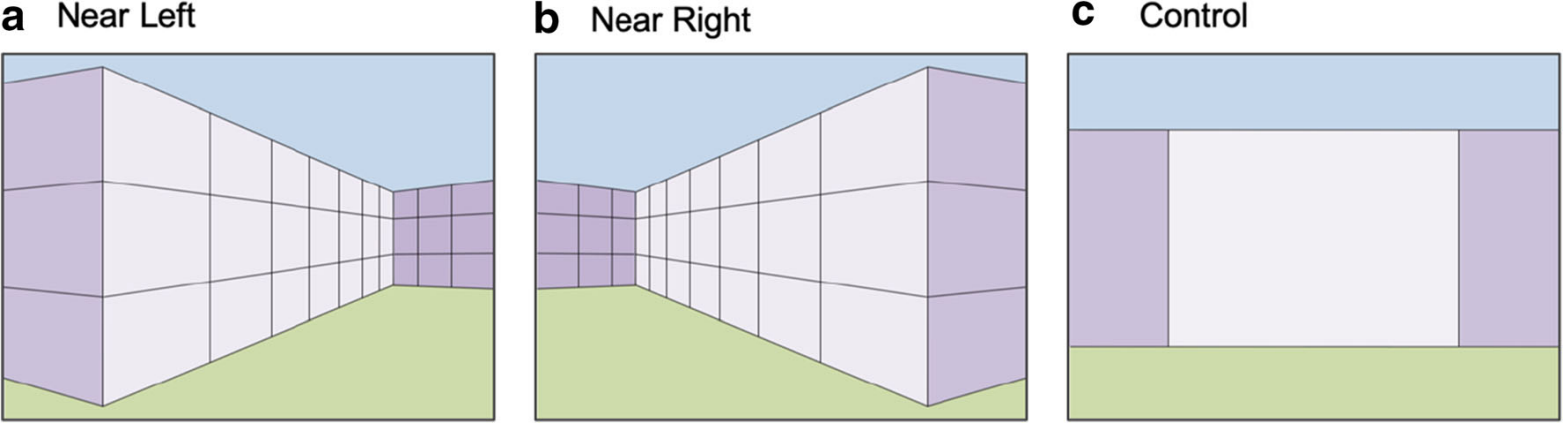
Ternus backgrounds. (**a**) and (**b**) Depth backgrounds vertically mirrored used for the Illusion Ternus task and the Illusion Magnitude task. (**c**) Control background used for Illusion Ternus task and Image Ternus task

In the size illusion illustrated in Fig. 1b, two stimuli of the same image size are perceived as different sized objects because they appear to be at different distances from the viewer within a depicted three-dimensional scene context. In particular, the stimulus that appears to be farther away from the viewer is perceived as larger than the stimulus that appears to be closer. This is consistent with the physics of three dimensions projecting onto two dimensions; an object will project a larger image onto a given projection plane when it is closer to that plane than when it is farther. Therefore, if an object that is (perceived as) farther away projects the same image size as one that is (perceived as) closer, it follows that the farther one is being projected by a larger object in the three-dimensional environment (e.g., Gregory, 2009; Rock, 1983). To investigate whether perceived size, beyond image size, determines correspondence, we presented identically sized Ternus displays on backgrounds (Fig. 3a and b; Illusion Ternus task; see video in Supplementary Online Material for an example) depicting depth such that they appeared to be either relatively near or relatively far from the viewer. If correspondence is based entirely on image size, then the perceived motion of the Ternus display should be unaffected by the apparent distance of the displays within these scenes. In contrast, if the perceived Ternus motion does vary with perceived distance, then we can infer that perceived size, which has to be abstracted from the size information that is directly available in the retinal image, contributes to the resolution of correspondence, and therefore that correspondence takes place at a higher level of visual processing than lower-level processes that extract the initial directly available retinal information. In particular it would be one at which the representation of relative depths of objects and perceived size has been established. In separate tasks within the experiment, we additionally measured the magnitude of the size illusion (Illusion Magnitude task) and then used Ternus displays with those physical sizes and presented them on a background without implied depth differences (Fig. 3c; Image Ternus task). This provided a direct comparison of the correspondence solution between perceived size differences and size differences that were explicit in the image.

## Method

### Participants

Twenty-four observers participated in the experiment (16 female, eight male, mean age = 24.04 years, *SD* = 3.13 years, range: 20–33). The sample size was calculated for an alpha of .05 with a power of .8 based on the effect size (partial eta square; Mordkoff, 2019) in a pilot study very similar to the Illusion Ternus task of this experiment. All observers were undergraduates from the University of Tübingen or from the surrounding community. They received 8 €/h or course credit in compensation for their time. All reported normal or corrected-to-normal visual acuity and were naïve as to the purpose of the experiment.

### Apparatus

The experiment was controlled by a Windows computer (Window XP) driving a 17-in. CRT color monitor with a spatial resolution of 1,024 x 768 pixels and a refresh rate of 100 Hz. MATLAB (Version R2012a, 7.14, Mathworks Inc., MA, USA) with Psychtoolbox 3 extensions (Brainard, 1997; Kleiner, Brainard, & Pelli, 2007; Pelli, 1997) was used to run the experiment. The experiment was conducted with the viewing distance fixed at 65 cm in a dimly lit individual testing room.

### Stimuli

In order to create the size illusion, a full-screen background was constructed that depicts a range of depths using linear perspective cues (see Fig. 3a and b). The depth-generating texture, i.e., a wall, was constructed in first-person perspective with two vanishing points and centered on the screen in a way that the observer perceived a nearest and a farthest point in the wall, which were equidistant from the center of the screen. Four different parts of the image were distinguished by their color: The upper part was blue (RGB: 185, 205, 229; 75 cd/m^2^), the lower part green (RGB: 195, 214, 155; 74 cd/m^2^), and the middle part with the wall texture was light purple (RGB: 230, 224, 236; 98 cd/m^2^) and dark purple (RGB: 179, 162, 199; 46 cd/m^2^), imitating the effect of an illumination source on the main part of the wall, the ends being in the shadow. Two different depth backgrounds were used (Near Left and Near Right, Fig. 3a and b) that were mirror versions of each other. The control background (Fig. 3c) was constructed to be as similar as possible to the depth background without using any perspective cues. The Ternus display (Pikler, 1917; Ternus, 1926) consisted of two frames with two elements vertically aligned with each other. By using the vertical version of the Ternus Display all elements were within the same perceived depth plane of the depth backgrounds and therefore perceived as being the same size. Each element had a diameter of 1.30° and the center-to-center separation between the elements was 1.63°. Depending on the presentation side, the Ternus display was presented 8.4° to the left or to the right of the screen center, the middle Ternus element across both frames vertically centered on the screen. The color of the Ternus elements was black (RGB: 0, 0, 0; 0 cd/m^2^) and the blank background between trials gray (RGB: 128, 128, 128; 20 cd/m^2^).

### Procedure

Participants were first informed about the experimental procedure and completed an informed consent process according to the ethical principles of the World Medical Association (World Medical Association, 2013). The experiment lasted about 60 min and included three subtasks: Illusion Ternus task, Illusion Magnitude task, and Image Ternus task. This order of the subtasks was the same across all participants. Each subtask started with written instructions on the screen.

**Fig. 4:**
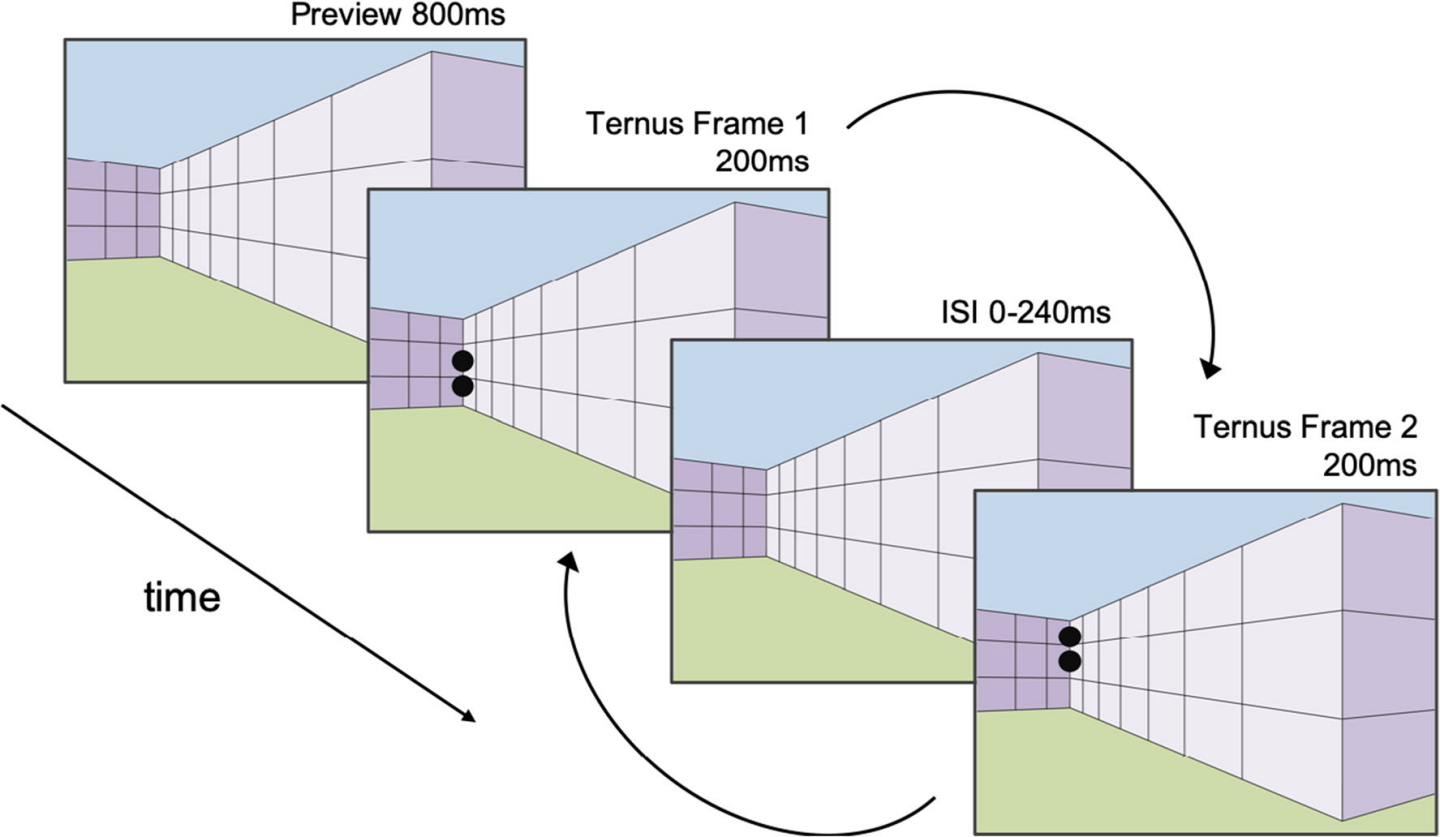
Illustration of the time course of a single Illusion Ternus task trial (shown here is the Near Right background with the Ternus presented on the left side, which corresponds to the perceived far distance)

For both the Illusion Ternus task and the Image Ternus task, participants were shown vertical versions of the Ternus display and asked to report whether they perceived element or group motion (see video in Supplementary Online Material for an example of the Illusion Ternus task). Following written instructions, demonstrations of clear element and clear group motion (using the most extreme ISIs of 0 and 240 ms, respectively) were presented. Participants performed a practice block of 18 trials and then completed six experimental blocks of 36 trials each. For the Illusion Ternus task in each trial (see Fig. 4), after a blank screen of 300 ms, one of the three different Ternus backgrounds (Near Left, Near Right, or Control; see Fig. 3) was presented for 800 ms, followed by the first Ternus frame superimposed on the background either in the left or right position for 200 ms. After a variable ISI of 0, 10, 20, 40, 80, or 240 ms, during which only the background was presented, the second Ternus frame was presented for 200 ms, followed by the background only for the same ISI. This cycle was repeated until participants responded.

For the Illusion Magnitude task, each trial started with a blank screen of 300 ms followed by one of the two depth backgrounds (Near Left or Near Right) for 800 ms. Then, Ternus elements were presented in each of the three positions of a Ternus display, in both the left and right positions of the background. Participants adjusted the size of this three-element Ternus display on either the left or the right side, which corresponded to the perceived near or far distance, depending on the depth background (Near Left or Near Right), until the displays on both sides were perceived as the same size. Which side was adjustable was chosen randomly. The non-adjustable three-element Ternus display (standard display) always had the size of 4.56° from edge to edge (element diameter: 1.30°; center-to-center separation between elements: 1.63°). Because both the individual elements as well as the space between them are perceived as changing in size in the Illusion Ternus task, each adjustment affected both the diameter of the elements and the space between the elements maintaining the proportions of the Ternus display. The start size of the adjustable three-element Ternus display was randomly either 1.47° larger or smaller than the size of the standard display (or 32.24% of the standard three-element Ternus display). Adjustment steps were around 0.06° for the entire three-element Ternus display.

The Image Ternus task used the values estimated from the Illusion Magnitude task to set display sizes. Procedurally, it was the same as the Illusion Ternus task, with the exception that only the control background was presented and not the depth backgrounds, and the Ternus display for a given trial was one of three different sizes (standard, small, and large), randomly selected for each trial. The standard display size was identical to that used in the Illusion Ternus task. The small display size corresponded to the individual estimated size of stimuli presented in the near distance in the Illusion Magnitude task. The large display size corresponded to the individual estimated size of stimuli presented in the far distance in the Illusion Magnitude task.

### Task

For both the Illusion Ternus task and the Image Ternus task participants reported whether the Ternus elements in the Ternus display appeared to be moving together (group motion) or as one element moving separately across the other element (element motion) by pressing the “J” or “F” key, respectively. In the Illusion Magnitude task participants adjusted the size of one of the two stationary columns of three-element display presented on the right and the left side until they perceived both as being the same size (method of adjustment; e.g., Coren & Girgus, 1972). The adjustments were made with the “J” (smaller) and “F” (bigger) key until the participants were satisfied with their result and confirmed with the space bar.

### Design

For the Illusion Ternus task a 6 (ISI: 0, 10, 20, 40, 80, 240 ms) x 3 (background: Near Left, Near Right, Control) x 2 (Ternus position: left, right) within-subject design was used. All factors were counterbalanced and randomly mixed within all trials. Each participant completed 216 trials, resulting in six observations per condition. For the Illusion Magnitude task, a 2 (background: Near Left, Near Right) x 2 (display adjustment side: left, right) within-subject design was used. All factors were counterbalanced and randomly mixed within all trials. Each participant completed four observations per condition. For the Image Ternus task a 6 (ISI: 0, 10, 20, 40, 80, 240 ms) x 3 (Ternus size: small, standard, big) x 2 (Ternus position: left, right) within-subject design was used. Again, all factors were counterbalanced and randomly mixed within all trials. Each participant completed six observations per condition.

## Results

Effect sizes are reported in terms of *adjusted partial eta-squared* ($adj\ {\hat{\eta}}_p^2$), which is an estimate of partial eta-squared that adjusts for the positive bias of the classic partial eta-squared that overestimates the population effect size (Mordkoff, 2019).

### Illusion Magnitude task

**Fig. 5:**
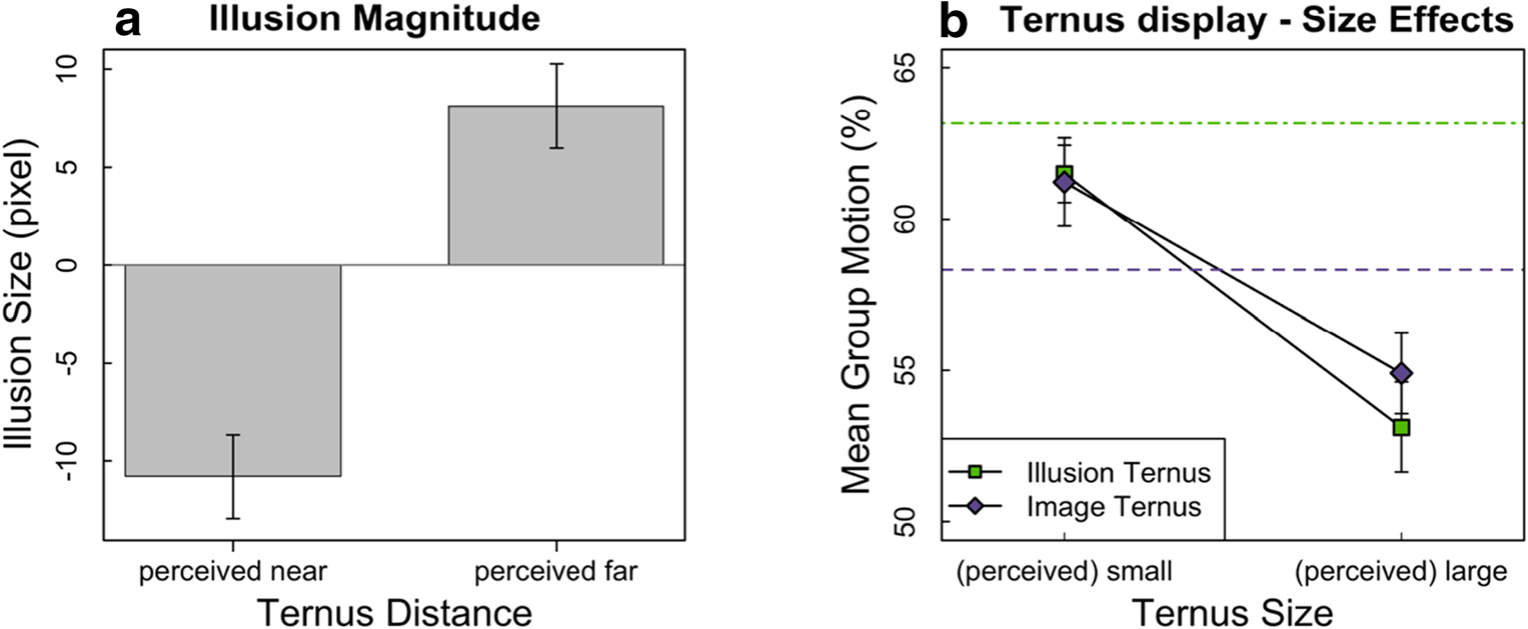
Results for all three experiments. (**a**) Illusion Magnitude. Perceived illusion size as a function of illusion condition (elements are perceived as smaller in the perceived near distance and as larger in the perceived far distance compared to the standard elements). (**b**) Effects of perceived and physical size in the Ternus display. Mean group motion responses as a function of the (perceived) small and large Ternus sizes for the Illusion and the Image Ternus task. The group motion responses for the baseline conditions (Image Ternus: standard Ternus size; Illusion Ternus: control background) are depicted with dotted and dot-dashed lines for the Illusion and the Image Ternus, respectively

To analyze the effect of the depth background on the perceived size of the Ternus elements we calculated the difference between the size of the standard Ternus elements and the size of the Ternus elements, which were adjusted by the participant. Negative values mean that the size of the adjusted elements was set to be larger than the standard size, and thus that the elements were perceived as smaller, while positive values mean that the size was set to be smaller than the standard size, and thus the elements were perceived as larger. As the two depth backgrounds were mirror versions of each other we combined the results from the adjusted elements based on their perceived distance (near or far). Figure 5A shows the mean perceived illusion size (in pixels; 1 pixel ≈ 0.02°) as a function of the perceived distance (near vs. far). Participants perceived the Ternus elements at the perceived near distance as significantly smaller (*mean element diameter* = 51.22 pixel; *SD* = 9.62 pixel) than elements at the perceived far distance (*mean element diameter* = 70.12 pixel; *SD* = 5.79 pixel), *t*(23) = -6.22, *p* < .001, $adj\ {\hat{\eta}}_p^2$=.61. In addition, one-sample *t-*tests revealed that both size percepts differed significantly from zero, with the Ternus elements in the perceived near distance perceived as smaller than the standard element, *t*(23) = -5.49, *p*_holm_ < .001, $adj\ {\hat{\eta}}_p^2$=.55, and the Ternus elements in the perceived far distance perceived as larger than the standard element, *t*(23) = 6.87, *p*_holm_ < .001, $adj\ {\hat{\eta}}_p^2$=.66.

### Illusion Ternus task

Next, we investigated whether the perceived size of the Ternus display can affect how correspondence is solved. Trials with responses other than the two response keys were excluded (0.96%) as well as trials with response times (RTs) longer than 8,000 ms (1.17%; mean RT: 1,799 ms). On the remaining trials we calculated the percent of group motion responses. Again, as the two depth backgrounds were mirror versions of each other, we combined the results from the left and right Ternus position based on their perceived distance. We also collapsed the results from the left and right Ternus position for the control background. This way, we created the new factor Ternus distance, with the levels perceived near, perceived far, and control distance. We conducted a 6 (ISI) x 3 (Ternus distance) repeated-measures ANOVA on the participants’ means of group motion percepts. The analysis revealed an effect of ISI, *F*(5,115) = 115.68, *p* < .001, $adj\ {\hat{\eta}}_p^2$ = .83, as group motion percepts were increasing with increasing ISI (from 6.11% at ISI 0 ms to 96.19% at ISI 240 ms), which is the effect typically observed in the Ternus display (e.g., Breitmeyer & Ritter, 1986a; Petersik & Pantle, 1979). Most importantly, there was a strong effect of Ternus distance, *F*(2,46) = 19.60, *p* < .001, $adj\ {\hat{\eta}}_p^2$ = .44 (Fig. 5b). Holms corrected post hoc comparisons revealed that significantly more group motion percepts were reported in the perceived near (*M* = 61.68%) compared to the perceived far condition (*M* = 53.50%), *t*(23) = 4.26, *p*_holm_ < .001, $adj\ {\hat{\eta}}_p^2$=.42, as well as between the control (*M* = 63.24%) and the perceived far condition, *t*(23) = 5.02, *p*_holm_ < .001, $adj\ {\hat{\eta}}_p^2$=.50, but no significant difference was found between the perceived near and the control condition, *t*(23) = -1.62, *p*_holm_ = .120, $adj\ {\hat{\eta}}_p^2$=.06. In addition, we found a significant interaction between ISI and Ternus distance, *F*(10,230) = 5.42, *p* < .001, $adj\ {\hat{\eta}}_p^2$ = .16. To investigate this interaction more closely, we conducted post hoc tests for each ISI between the two Ternus distance conditions that differed from each other (perceived far and near distance). They revealed significant and marginally significant differences for the ISI with the most ambiguous percept, i.e., the 10 and the 40 ms ISI, 3.94 <= *t*(23) <= 4.89, *p*_holm_ <= .003, $adj\ {\hat{\eta}}_p^2$<= .49, and the ISI of 20 ms, *t(23)* = 2.48, *p*_holm_ = .083, $adj\ {\hat{\eta}}_p^2$<= .18, but no significant differences for the less ambiguous ISI conditions, i.e., the 0, the 80, and the 240 ms ISI, 0.35 <= *t*(23) <= 0.96, *p*_holm_ = 1, $adj\ {\hat{\eta}}_p^2$<=-.003.

### Image Ternus task

Finally, we examined whether a physical size difference comparable to the individually perceived size difference obtained in the Illusion Magnitude task had a similar effect on the motion percept in the Ternus display. Trials with responses other than the two response keys (0.95%) and trials with RTs longer than 8,000 ms were excluded (0.78%, mean RT: 1,659 ms). Again, we combined the results from the left and right Ternus position. Therefore, we performed a 6 (ISI) x 3 (Ternus size) repeated-measures ANOVA on the mean percent of group motion percepts. There was again the typical ISI effect, *F*(5,115) = 74.62, *p* < .001, $adj\ {\hat{\eta}}_p^2$=.75, as mean group motion responses increased with increasing ISI (from 6.67% at ISI 0 ms to 94.08% at ISI 240 ms). Most importantly, Ternus size influenced motion perception, *F*(2,46) = 6.44, *p* = .007, $adj\ {\hat{\eta}}_p^2$=.18 (Fig. 5b). Holm’s corrected *t*-tests for each Ternus size condition showed significantly less group motion percepts for the large (*M* = 54.91%) compared to the standard Ternus size (*M* = 58.32%), *t*(23) = -2.46, *p*_holm_ = .044, $adj\ {\hat{\eta}}_p^2$=.18, and compared to the small Ternus size (*M* = 61.22%), *t*(23) = -2.92, *p*_holm_ = .023, $adj\ {\hat{\eta}}_p^2$=.24. There was no significant difference between the small and the standard Ternus size, *t*(23) = 1.77, *p*_holm_ = .090, $adj\ {\hat{\eta}}_p^2$=.08. In addition, there was a trend for an interaction between ISI and Ternus size, *F*(10,230) = 2.12, *p* = .058, $adj\ {\hat{\eta}}_p^2$ = .04. To investigate this trend more closely, we conducted post hoc tests for each ISI between the two significantly different Ternus size conditions (small and large Ternus size). They revealed significant differences for the most ambiguous ISI condition of 20 and 40 ms, 3.21 <= *t*(23) <= 3.34, *p*_holm_ <= .020, $adj\ {\hat{\eta}}_p^2$<= .30, but no significant differences for the other ISI, 0.12 <= *t*(23) <= 2.28, *p*_holm_ >= .129, $adj\ {\hat{\eta}}_p^2$<=.15.

A notable aspect of the results is that the impact of size on perceived Ternus motion appears to be nearly the same whether the size differences are illusory (Illusion Ternus task) or physical (Image Ternus task). To assess this, we conducted an additional post hoc 2 Task x 2 Ternus Size x 6 ISI repeated-measures ANOVA. Results showed no main effect of the factor task, *F*(1,23) = 0.04, *p* < .849, $adj\ {\hat{\eta}}_p^2$ = -.04. We also found no interaction between the factors task and size, *F*(1,23) = 0.41, *p* < .529, $adj\ {\hat{\eta}}_p^2$ = -.03, mean group motion responses being similar for the perceived small (61.68%,) and the physically small condition (61.22%), as well as for the perceived large (53.50%) and the physically large condition (54.91%). In addition, no other interactions with the factor task were significant, 0.87 <= *F* <= 2.13, *p* <= .091, $adj\ {\hat{\eta}}_p^2$<=.05.

## Discussion

A critical function of vision is to establish and maintain representations of objects that have continuous identities over space and time, even as retinal input changes or disappears. An important aspect of achieving that function is determining which stimuli across time and space correspond to the same or different objects, a problem known as the correspondence problem (Ullmann, 1979). Previous work has shown that feature information at the level of the retinal image plays an important role in how the correspondence problem is solved by the visual system (e.g., Breitmeyer & Ritter, 1986a, 1986b). In this study, we investigated whether it is feature information at the level of the retinal image or at the level of the perceived object that is critical for the correspondence process. We found that the perception of Ternus motion varied with feature information at the level of the perceived object, more precisely with the perceived size of the stimuli evoked by different illusory depth backgrounds. In a separate task, we measured the magnitude of the size illusion and confirmed that the elements in the Ternus displays that appeared to be at the farther distance in the illusory depth scene were perceived as larger than those that appeared to be at the nearer distance. Finally, we used those measured magnitudes to create Ternus displays with corresponding *physical* size differences (all presented at the same apparent distance from the viewer), and found that the differences in perceived Ternus motion for the physically different stimuli matched the differences for the perceptually different, but physically identical, stimuli. Together, these results provide strong evidence that the correspondence process is resolved on the basis of higher-level properties of represented objects, rather than on lower-level properties of the image input.

The finding that larger Ternus displays, whether physically larger or illusorily larger, lead to less group motion percepts may appear contrary to studies that have found *more* group motion reports for physically larger Ternus elements (Breitmeyer & Ritter, 1986a, 1986b; Casco, 1990, Exp. 4). However, in those studies, the size of the individual elements was manipulated without changing the center-to-center separation between elements. A consequence of this is that the edge-to-edge separation between elements decreased with increasing element size and increased with decreasing element size. Larger edge-to-edge separation, however, is known to yield less group motion percepts (Pantle & Petersik, 1980). Furthermore, when element size and element separation were manipulated factorially, element separation was found to be the more important factor for determining correspondence (Casco, 1990; Petersik & Grassmuck, 1981). In the current study, size manipulations were designed to mimic metric size changes in image projections. Therefore, the ratio between the size of the elements and the distance was held constant across separation conditions, i.e., a change in element size included a corresponding change of the separation between elements. The pattern of our results, therefore, do not conflict with those of previous studies, but rather fit well with them.

Because perceived size depends on perceived depth, the fact that Ternus motion depended on perceived size further indicates that correspondence is resolved after depth information is encoded. This follows because the size illusion depends on the Ternus displays being perceived as being at different distances from the viewer as supported by the pictorial depth cues in the background displays (Gregory, 2009; Rock, 1983). The displays used in this study were inspired by the standard Ponzo illusion, which includes only two converging lines. Early considerations of that simpler illusion included the possibility that it was driven by lower-level image characteristics, rather than higher-level implications of depth relations (see Prinzmetal, Shimamura, & Mikolinski, 2001). But there is recent evidence that even simpler versions depend on higher-level information. Brown, Breitmeyer, Hale, and Plummer (2018) measured the contrast response function (CRF) for the traditional simple Ponzo illusion, i.e., how the magnitude of the illusion changes as a function of the contrast of inducing stimuli (i.e., the converging lines). They found non-linear changes in the CRF for the Ponzo illusion, indicating a dependence on higher-level perceptual coding (e.g., perceived size and distance). The authors therefore assumed that the Ponzo illusion involves higher-level information, dependent on representations in cortical regions, like V4, LOC, and inferotemporal cortex. The current results showed that Ternus motion depended on perceived depth and size, therefore suggesting that correspondence can happen at least at these levels of processing.

Finally, the current results are also consistent with previous studies showing that amodally completed stimuli play a role in how apparent motion is perceived (e.g., Hein & Moore, 2014). That work emphasized the conclusion that correspondence in motion perception depends not only on low-level motion energy (Adelson & Bergen, 1985; van Santen & Sperling, 1985; Werkhoven, Sperling, & Chubb, 1993), but also on higher-level perceptual representation in which information about the structure and the content of the environment has been abstracted from the initial image-level input. The current results reinforce this conclusion by showing that the influence of object-based information occurs at or beyond the level of amodal completion, at which image-based information was further processed taking into account context information. Therefore, this study offers further support for an object-based correspondence theory (Hein & Cavanagh, 2012; Hein & Moore, 2014; Stepper, Moore, Rolke, & Hein, 2019; Stepper, Rolke, & Hein, 2019), which states that all available information about an object, low-level and high-level, is taken into account for establishing correspondence, based on the (perceived) similarity between the individual element across frames.

In summary, using Ternus displays in the context of a depth-based size illusion, we found that the perceived size of objects, not simply image size, determines how correspondence is established. This indicates that the correspondence process takes place after pictorial cues are used by the visual system to establish representations of depth relations and structure.

## Electronic supplementary material

ESM 1 (MOV 27 mb)
